# The response of correlated traits following cessation of fishery-induced selection

**DOI:** 10.1111/j.1752-4571.2012.00243.x

**Published:** 2012-11

**Authors:** Santiago Salinas, Kestrel O Perez, Tara A Duffy, Stephen J Sabatino, Lyndie A Hice, Stephan B Munch, David O Conover

**Affiliations:** 1 School of Marine and Atmospheric Sciences, Stony Brook UniversityStony Brook, NY, USA; 2 Department of Ecology and Evolution, Stony Brook UniversityStony Brook, NY, USA; 3 National Marine Fisheries ServiceSanta Cruz, CA, USA; 4 Division of Ocean Sciences, National Science FoundationArlington, VA, USA

**Keywords:** experimental evolution, fisheries management, life history evolution, wildlife management

## Abstract

The application of evolutionary principles to the management of fisheries has gained considerable attention recently. Harvesting of fish may apply directional or disruptive selection to key life-history traits, and evidence for fishery-induced evolution is growing. The traits that are directly selected upon are often correlated (genetically or phenotypically) with a suite of interrelated physiological, behavioral, and morphological characters. A question that has received comparatively little attention is whether or not, after cessation of fishery-induced selection, these correlated traits revert back to previous states. Here, we empirically examine this question. In experiments with the Atlantic silverside, *Menidia menidia*, we applied size-selective culling for five generations and then maintained the lines a further five generations under random harvesting. We found that some traits do return to preharvesting levels (e.g., larval viability), some partially recover (e.g., egg volume, size-at-hatch), and others show no sign of change (e.g., food consumption rate, vertebral number). Such correlations among characters could, in theory, greatly accelerate or decelerate the recovery of fish populations. These results may explain why some fish stocks fail to recover after fishing pressure is relaxed.

## Introduction

Recent theoretical and empirical research has shown the importance of incorporating evolutionary theory into fisheries management (Law 2007). Size-selective mortality can be a strong human-induced evolutionary force, and the harvest of fishes provides a particularly striking example (Allendorf and Hard 2009). Fishing mortality is often a strong size-selective pressure that can exceed natural mortality by as much as 400% (Mertz and Myers 1998). In addition, the heritability of life-history traits, such as age-at-maturity, is relatively high [mean of 8 studies = 0.31, SD = 0.19 (Law 2000)]. Studies have demonstrated the rapid evolution of traits that are of direct relevance to fisheries, including body size, growth rate, and age-at-maturation (reviewed in Jørgensen et al. 2007). It is, therefore, crucial to investigate how fishing pressure will alter the evolutionary trajectory of natural populations.

Fishing indirectly selects for life-history traits [e.g., growth rate (Law and Rowell 1993)] that are often genetically or phenotypically linked to other traits. Fisheries-induced selection may, therefore, result in changes in multiple traits, which may collectively cause substantial reductions in the resilience and health of fish stocks (Hutchings 2005). In an experiment designed to simulate fishing by applying strong size selectivity over five generations in *Menidia menidia*, Conover and Munch (2002) showed that body size and growth rate can evolve rapidly. Using fish from the same study, Walsh et al. (2006) showed that selection on adult size can lead to significant changes in other physiological, morphological, developmental, and behavioral traits that depress fitness relative to an unfished stock. The importance of correlated characters in harvest selection has been abundantly documented. Foraging behavior (Biro et al. 2004; Chiba et al. 2007), smolting (Thrower et al. 2004), sex change (Walker et al. 2007), and other traits appear to share genetic and/or phenotypic covariances with other traits that are subject to fisheries-induced selection. Furthermore, Hard (2004) showed that, in chinook salmon (*Oncorhynchus tshawytscha*), accounting for correlated characters in a multivariate quantitative genetic model yielded responses to selection much greater than estimates based on single-trait approaches.

Because evolutionary responses to fishing have the potential to negatively affect population productivity, it is crucial to know whether such changes – both direct and indirect – are easily reversed. Theoretical studies have focused on traits directly impacted by fisheries and suggested that recovery may be slow, if at all possible [e.g., (Hutchings 2005)]. However, Conover et al. (2009) recently provided evidence from laboratory experiments for the partial recovery after selection is relaxed: in the continuation of the experiment mentioned previously, generations 6–10 of *M. menidia* were harvested randomly, and the final populations displayed a partial recovery, at least for size-at-age (Conover et al. 2009). These results are consistent with work on wild populations. For example, growth in Lake Windermere pike (Edeline et al. 2007) appears to rebound in response to reductions in fishing pressure.

Given that fishing greatly alters a suite of correlated characters (Walsh et al. 2006), would the cessation of fishing allow the entire set of traits to be reconstituted just as they were before selection? No empirical data currently exist to address this question. Here, we take advantage of the long-term *M. menidia* experiments to assess the capacity for traits that evolved, but were not directly selected for, to return to preharvesting levels.

## Methods

The experimental fish populations used in this study originate from an 11-year fishery simulation study started in 1998 (Conover and Munch 2002). Six wild populations of *M. menidia* from New York were initially divided into three treatments: small-, random-, and large-harvested. In the large-harvested populations (*n* = 2), fish were measured on day 190, and the smallest 10th percentile were allowed to spawn, while in the small-harvested populations (*n* = 2), the largest 10th percentile were used to breed the next generation. These were compared with random-harvested control populations (*n* = 2). This was carried out for five generations while assessing growth rate and individual body weight at days 90 and 190 postfertilization (Conover and Munch 2002). Additionally, multiple characters related to growth and reproduction were assessed on fish from generation 5 (Walsh et al. 2006). Following generation 5, harvesting was applied randomly to all treatments for an additional five generations (Conover et al. 2009). During this time, all six populations experienced the same laboratory and feeding conditions. Full experimental details can be found elsewhere (Conover and Munch 2002; Walsh et al. 2006; Conover et al. 2009).

In this study, a variety of traits were measured in fish of generation 11. All the traits investigated here have been shown to be genetically correlated with growth rate or size-at-age in fish species [egg volume (Su et al. 2002), size-at-hatch and larval viability (Munch et al. 2005), vertebral number (Ando et al. 2008), growth efficiency (Henryon et al. 2002), food consumption rate (Kause et al. 2006)]. Thus, it is instructive to examine how these correlated characters evolve in response to fishery-like selection and moratoria.

To allow for comparisons with data from generation 5, the methods followed by Walsh et al. (2006) were closely replicated. They are briefly described below:

### Egg volume

Thirty plus eggs from each line for three separate spawning events were photographed and measured digitally. Eggs were assumed spherical for volume calculations.

### Larval size at hatch

Thirty 1-day-old larvae per population were measured over five different hatch dates.

### Larval viability

Viability, defined here as the proportion of larvae surviving to day 10, was measured with fish from three different hatch dates. Fifty larvae from each of the six populations were placed in buckets at 21°C and fed *ad libitum*; survivors were then counted at day 10.

### Food consumption and conversion efficiency

To evaluate food consumption, two sets of six size-matched fish per selected line were separated. One set was offered unlimited food (*Artemia* nauplii) daily throughout 10 days, while the other was offered a limited ration equivalent to 50% wet fish mass. Daily, before feeding, uneaten food was collected and weighed. At day 10, all fish were measured for length, wet weight, and dry weight. Daily mean food consumption (mg day^−1^) was calculated as (food offered – food retrieved) / (# fish x # days). This was repeated with fish from three different hatch dates. Growth efficiency (%) was calculated as the total increase in dry weight divided by the total dry weight consumption of brine shrimp.

### Vertebral number

Adult fish from breeding populations in generation 10 were X-rayed on Kodak Industrex MX125 film using a Hewlett Packard Faxitron X-ray system. Films were manually developed, and fish were scored for vertebral number under a microfilm machine. Counts were made of the centra between the basioccipital and urostyle. Any fish with clear vertebral deformities, such as fused vertebrae, were removed from the analysis.

Full recovery of a trait would be evidenced by similar values of that trait among small-, random-, and large-harvested treatments in generation 11. To compare the three treatments in generation 11, we used Kruskal–Wallis anova because of non-normality in the data. To assess partial recovery, comparisons of slopes (of trait value against treatment) between generation 5 and generation 11 were conducted via generalized linear models. Specifically, we used the significance of the generation-by-treatment term as an indicator of partial trait reversal. In a generalized linear model, 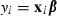 where *y*_*i*_ is the dependent variable, 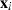 is a vector of predictors (generation, selection line, and interaction), and 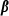 is a vector of unknown parameters. The linear predictor is a function of the mean parameter via a link function, which can take many forms depending on the distribution of data. We used generalized linear models with a gamma distribution and a log link function for egg volume, larval size at hatch, food consumption, and conversion efficiency. Larval viability data were tested with a generalized linear model with a binomial distribution and a logit link function. The generalized linear model for vertebral number assumed a Poisson distribution and a log link function. Replicates were nested within treatment lines in all cases, except for the food consumption and conversion efficiency experiments in generation 11 (space constraints only allowed for the testing of one line).

The degree of recovery for each trait can be summarized with an index of recovery that accounts for sampling variability calculated as


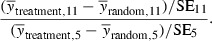


A value of one indicates that the trait showed no difference when compared to the random-size harvested populations after five generations of random selection, while a 0 represents perfect recovery. A negative value, in turn, describes overcompensation in the response.

## Results

### Full recovery

**Figure 1:**
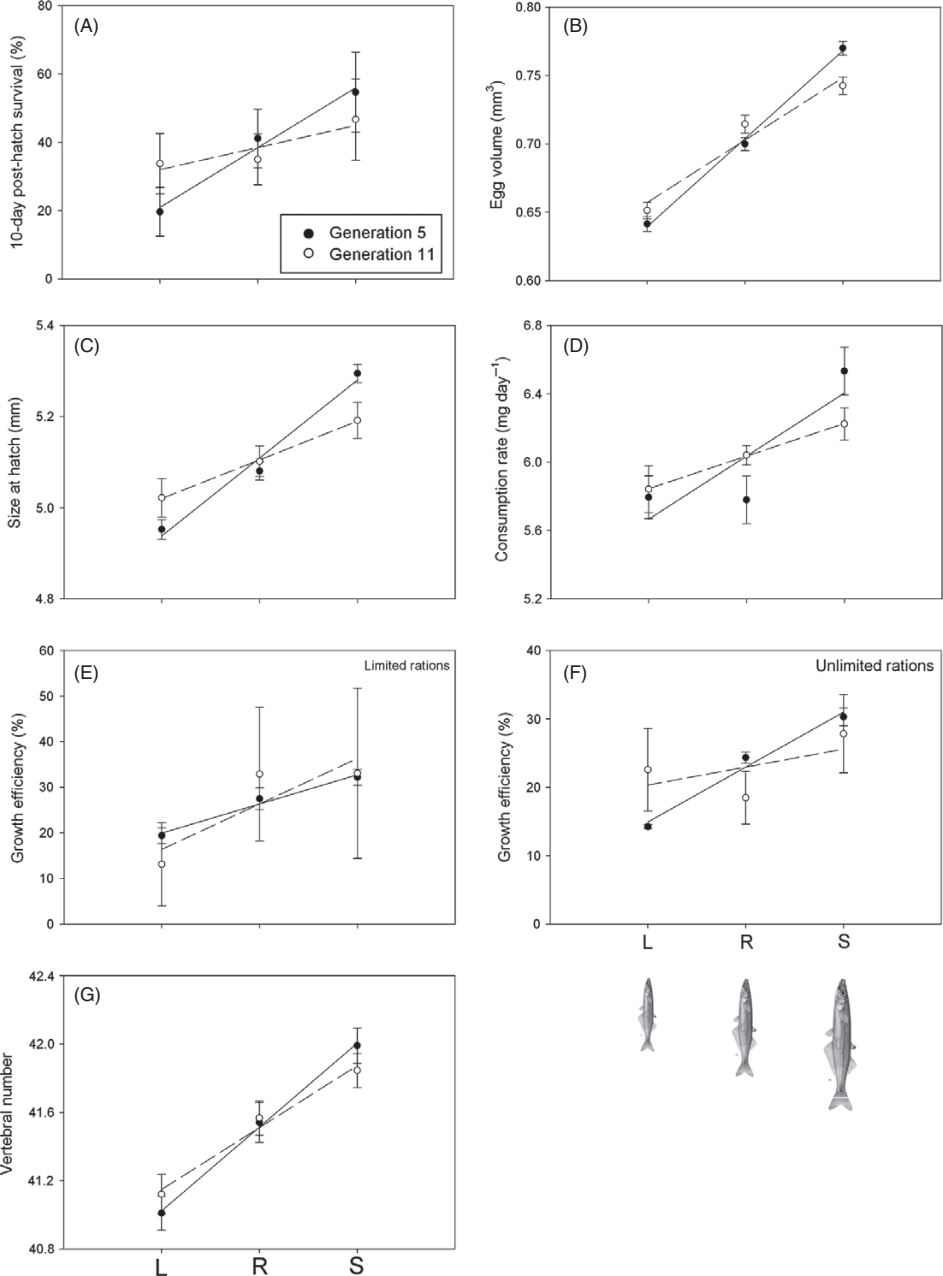
Adjusted means (calculated as 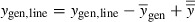) ±1 SE for large-size harvested (L), random-size harvested (R), and small-size harvested lines (S) in generation 5 (black) and generation 11 (white). *y*_gen,line_ is the mean of a line (L, R, S) from a given generation (5, 11), 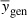 is the mean trait value for that generation, and 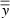 is the grand mean trait value (across generations and treatments). The traits are (A) larval viability, (B) egg volume, (C) larval size at hatch, (D) consumption rate under unlimited food conditions, (E) growth efficiency under restricted food conditions, (F) growth efficiency under unlimited food conditions, and (G) vertebral number. Note that the lines are for visualization purposes only.

Larval viability apparently recovered after a period of random selection (GLM generation-by-treatment interaction *P* < 0.001; Fig. 1A, Table 1). The rebounding was pronounced, and differences among selection lines in generation 11 were small and nonsignificant (Kruskal–Wallis anova: *H* = 1.172, *P* = 0.557).

**Table 1:** Recovery index of the seven measured traits in small-size and large-size harvested populations. The smaller the index value, the more of a rebound the trait exhibited. The index was calculated as 
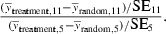

	Recovery index	
	
Trait	Small-size harvested	Large-size harvested
Larval viability	0.928	0.061
Egg volume	0.356	0.956
Size-at-hatch	0.242	0.358
Consumption rate	0.364	−19.997
Growth efficiency (LIM)	0.003	0.184
Growth efficiency (UNL)	0.323	−0.083
Vertebral number	0.644	0.877

### Partial recovery

Egg volume showed some signs of recovery (GLM generation-by-treatment *P* < 0.001; Fig. 1B, Table 1). The recovery, however, was not fully realized. Egg volume remained different for the three selection lines at generation 11 (Kruskal–Wallis anova: *H* = 104.313, *P* < 0.001). Eggs in the small-harvested populations were 13% larger than those from their large-harvested counterparts (pairwise multiple comparisons indicated that large-, random-, and small-harvested populations’ egg volumes were all significantly different from each other).

Size-at-hatch also showed signs of return to preharvest levels (GLM generation-by-treatment *P* < 0.001; Fig. 1C, Table 1). Differences between lines were much smaller than those observed in generation 5 (small-harvested populations were 6.8% larger than large-harvested ones in population 5 and 3.2% larger in generation 11). Similar to egg volume, although, the rebounding over the time period studied was incomplete (GLM generation-by-treatment *P* < 0.001). Significant differences in size-at-hatch remained in generation 11 among large-, random-, and small-harvested populations (*H* = 10.385, *P* = 0.006; pairwise multiple comparisons indicated that only differences between large- and small-harvested were significant).

### No recovery

Food consumption rate, conversely, did not exhibit a recovery trend (Fig. 1D, Table 1). Generation 5 and generation 11 differences among treatments remained similar (GLM generation-by-treatment *P* = 0.090). Fish from large- and random-harvested populations in generation 11 consumed food at a slightly lower rate than those from small-harvested populations (*H* = 5.067, *P* = 0.080).

Interestingly, whether or not growth efficiency rebounded was determined by the amount of food available. When fed a limited ration, *M. menidia*’s growth efficiency did not show signs of recovery (GLM generation-by-treatment interaction *P* = 0.960; Fig. 1E, Table 1), although the results were highly ambiguous, as the different selection lines were equally efficient at converting food into growth (*H* = 1.818, *P* = 0.561). On the other hand, when on an unlimited diet, recovery of growth efficiency did occur (GLM generation-by-treatment interaction *P* = 0.039; Fig. 1F, Table 1). The return to preselection levels appears complete, as there were no differences in efficiency between selection treatments (*H* = 2.222, *P* = 0.329).

Vertebral number also failed to revert back to preselection levels (GLM generation-by-treatment interaction *P* = 0.981; Fig. 1G, Table 1). In generation 11, significant differences among the treatments remained (*H* = 20.892, *P* < 0.001). These were due primarily to the large-harvested populations, which had fewer vertebrae than either the random- or small-harvested populations.

## Discussion

In our experimental fishery, many traits that were not directly selected upon experienced large changes during the 5-generation fishing period (Walsh et al. 2006). After a subsequent five generations of random harvesting, during which growth rate and body size partially rebounded (Conover et al. 2009), the correlated traits did not recover uniformly. Larval viability and growth efficiency under unlimited rations returned to preharvesting levels. Partial rebounding was found in egg volume and size-at-hatch. No signs of recovery were seen in food consumption rate, vertebral number, and growth efficiency under limited rations. It must be noted, however, that the higher SE in the growth efficiency trials in generation 11 (particularly in the limited ration ones) relative to generation 5 makes recovery assessments difficult.

What drives these disparate responses? During the initial period of selective fishing, the main traits under selection were adult size and growth rate. After generation 5, fishing was randomized with respect to size, and the primary traits under selection shifted. All else being equal, within each line, females with larger egg clutches would be expected to produce a greater fraction of the larval pool that was reared to adulthood. Variation in the viability of these larvae, particularly with respect to early feeding success, is also likely to be a source of selection in the experiment. Consequently, characters correlated with fecundity and early survival would exhibit a return to preharvesting levels. It is then no surprise that 10-day posthatch larval viability fully reverted back in six generations. Our results, therefore, support previous assertions that changes in the selective landscape when fishery mortality is relaxed (Edeline et al. 2007; Swain 2011) will influence which traits rebound to preharvesting levels.

It is worth noting that there was substantial variation in the grand mean across generations for nearly all of the traits (Figure S1). This variation may result from plastic responses to unintentional variation in the laboratory environment as well as domestication selection. The importance of maintaining unselected controls (Falconer and Mackay 1996) is, therefore, critical to the interpretation of selection experiments. However, all treatments in our experiment (small-, random-, and large-harvested lines) experienced the same laboratory conditions and selective environment, and it is, thus, expected that all shared the same phenotypic optimum. Therefore, as our interest lies in evaluating recovery from an earlier period of selection, it is the reduction in variation among lines that is most relevant.

All of the traits studied here are relevant to the health of fish stocks and may impact fishery yields. For example, egg size, size-at-hatch, and larval survival can have important effects on recruitment and population dynamics (Houde 1989; Pepin and Myers 1991), while consumption rate and growth efficiency could influence survival independently of growth (Bochdansky et al. 2008).

Results across the emerging field of Darwinian fisheries science indicate that evolution of characters during periods of intense harvesting may be relatively easy to predict. The pace and direction of evolution in response to traits directly selected by harvesting have closely agreed with theoretical expectations (Law 2007; Hard et al. 2008). Moreover, the response of correlated traits found by Walsh et al. (2006) in *M. menidia* mimics those found across latitudinally distinct populations in the wild (Conover et al. 2005), suggesting that among-population covariances inferred from locally adapted populations may be used to predict how multiple traits will evolve in response to fishing pressure.

Once fishery selection is reduced, however, our results indicate that not all traits revert back to the previous state. Even after the focal trait (e.g., size-at-age) has recovered (Edeline et al. 2007; Conover et al. 2009), the population is not the same, and some postselection traits may continue to be expressed. As the recovery of a trait to preselection levels depends on how closely it is tied to fitness (Estes and Teotónio 2009), knowledge of genetic correlations (Hutchings and Fraser 2008) alone will be insufficient to predict recovery. Knowledge of the natural adaptive landscape is also required. In addition, the genetic architecture of the population (Jeffery 2001) and previous evolutionary history (Teotónio et al. 2002) may also affect recovery. In light of this, forecasting patterns of recovery may be considerably more complicated than predicting the initial response to harvest and is unlikely to be feasible for many populations. A precautionary approach to management would, therefore, attempt to minimize selection imposed by harvesting.

## Data archiving statement

Data deposited in the Dryad repository: doi: 10.5061/dryad.4k24d37q.

## Supporting Information

**Figure S1.** Traits' raw means.
