# Supplementary material for: The response of correlated traits following cessation of fishery-induced selection
Source: Evol Appl. 2012 Nov;5(7):657–63. doi: 10.1111/j.1752-4571.2012.00243.x (PMC3492892; doi:10.1111/j.1752-4571.2012.00243.x)

**Supporting Information**

**Figure S1 – Traits’ raw means**

Means ±1 SE for large-size harvested (L), random-size harvested (R), and small-size harvested lines (S) in generation 5 (black) and generation 11 (white). The traits are (a) larval viability, (b) egg volume, (c) larval size at hatch, (d) consumption rate under unlimited food conditions, (e) growth efficiency under restricted food conditions, (f) growth efficiency under unlimited food conditions, and (g) vertebral number. Note that the lines are for visualization purposes only.


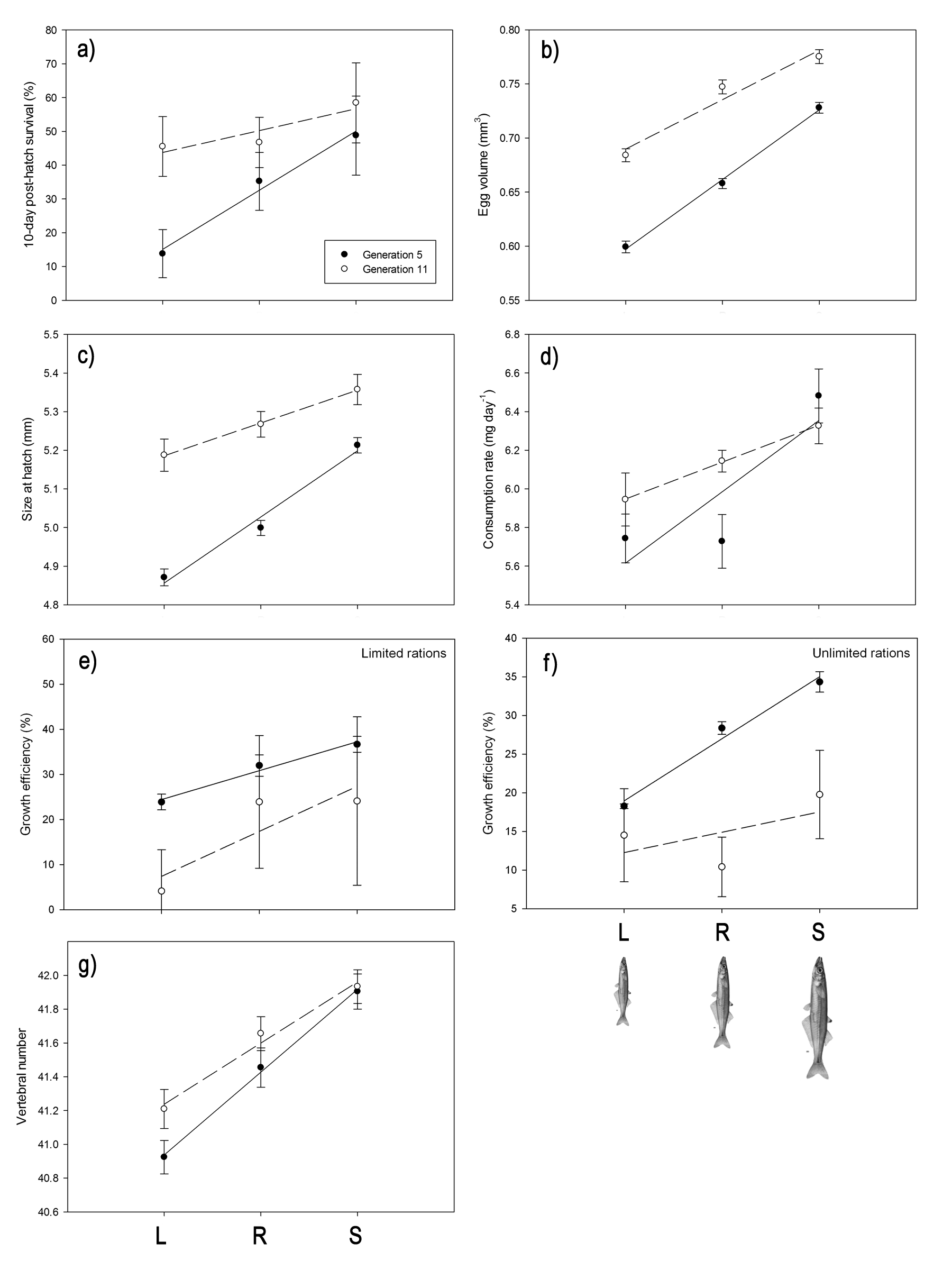

Supplement: Supplementary file 1 [file eva0005-0657-SD1.doc]
